# Atomistic switch of giant magnetoresistance and spin thermopower in graphene-like nanoribbons

**DOI:** 10.1038/srep36762

**Published:** 2016-11-18

**Authors:** Ming-Xing Zhai, Xue-Feng Wang

**Affiliations:** 1 Jiangsu Key Laboratory of Thin Films, College of Physics, Optoelectronics and Energy, Soochow University, 1 Shizi Street, Suzhou 215006, China; 2 Hongzhiwei Technology Co. Ltd., 1888 Xinjinqiao Road, Pudong, Shanghai 201206, China; 3 Key Laboratory of Terahertz Solid-State Technology, Shanghai Institute of Microsystem and Information Technology, Chinese Academy of Sciences, 865 Changning Road, Shanghai 200050, China

## Abstract

We demonstrate that the giant magnetoresistance can be switched off (on) in even- (odd-) width zigzag graphene-like nanoribbons by an atomistic gate potential or edge disorder inside the domain wall in the antiparallel (*ap*) magnetic configuration. A strong magneto-thermopower effect is also predicted that the spin thermopower can be greatly enhanced in the ap configuration while the charge thermopower remains low. The results extracted from the tight-binding model agree well with those obtained by first-principles simulations for edge doped graphene nanoribbons. Analytical expressions in the simplest case are obtained to facilitate qualitative analyses in general contexts.

The giant magnetoresistance (GMR) effect, which is discovered in sandwiched structures of magnetic and nonmagnetic materials in 1988^1^,^2^, means that a large conductance difference turns up when the relative spin orientation in adjacent magnetic materials change from parallel (*p*) to antiparallel (*ap*). GMR is essential in some spintronic devices that manipulate electron spin rather than charge^3^ and has led to the explosive enlargement of storage capability on hard disk in the last decades, reflecting its important value in commercial applications.

Charge and spin thermopower of a material describes the ability to produce charge and spin current, respectively, from a temperature gradient rather than a voltage one. Charge thermopower devices such as thermocouple have been widely used in our daily life. Spin thermopower has been observed in magnetic^4^ and nonmagnetic^5^ materials and should have application potential in spintronics. Similar to the GMR effect, the thermopower in magnetic junctions can change when the magnetic configuration changes from *p* to *ap* and lead to the magneto-thermopower phenomena^6^. The GMR and thermopower effects are both intrinsically relevant to the transmission spectrum in tunneling junctions. Recently, magnetoresistance^7^,^8^,^9^,^10^,^11^,^12^,^13^ and spin thermopower^13^,^14^,^15^,^16^ in graphene-like nanoribbons have become one of the focuses.

The discovery of graphene and its many outstanding properties^17^ has inspired enormous attentionon on graphene-like two-dimensional (2D) materials. The long spin relaxation time and length in graphene indicates it a prospective material for spintronics^18^. Specially, experimental observations have confirmed the existence of magnetism on zigzag edges of graphene^19^,^20^. Both theoretical models^20^,^21^,^22^ and computational simulations based on *ab initio* density functional theory^23^,^24^ have shown that the magnetism origins from the spin polarized edge atoms. Zigzag graphene nanoribbons (ZGNRs) have two zigzag edges and can be in ferromagnetic (FM) or antiferromagnetic (AFM) state classified by the relative spin orientations on the two edges^20^,^24^. External magnetic field can convert a ZGNR from its ground insulator AFM state to its metallic FM state. The consequent large colossal magnetoresistance was observed persistent up to room temperature^7^. In addition, GMR in FM ZGNRs of even width has been predicted very high^10^ as well as the colossal magnetoresistance^8^. ZGNR based GMR devices are believed very useful based on the fact that ZGNRs can be synthesized and relevant devices fabricated in atomic precision with the state-of-the-art technology^19^,^20^. Traditional chemical and physical edge functionalizations in atomistic scale such as atom adsorption, doping, vacancy and local lattice distortion have been proposed to manipulate further the properties and design diversified devices^25^,^26^. Specifically, the edge disorders or defects can break geometry symmetry of ZGNRs and enhance the spin thermopower effect. In addition, scanning tunneling microscopy (STM) tip and atomic force microscopic techniques can be used as atomistic gate to apply local electrostatic potential for graphene transistors and manually introduce and control the disorders^27^,^28^. In this work, based on the tight-binding model and the first-principles simulations, we propose a mechanism to switch on and off GMR and spin thermopower by applying an atomistic extrinsic potential on edge of FM zigzag nanoribbons (ZNRs) of two-dimensional (2D) graphene-like honeycomb lattice. Our prediction may be confirmed via an experimental setup schemed in Fig. 1 by combining the techniques for measuring the current-voltage curve of narrow nanoribbons^7^,^29^,^30^ with the STM techniques for controlling the potential in atomistic scale^27^,^28^. In addition, the effects discussed in the following for single local potential or impurity might be enhanced in disordered systems^31^.

## Models and Methods

**Figure 1:**
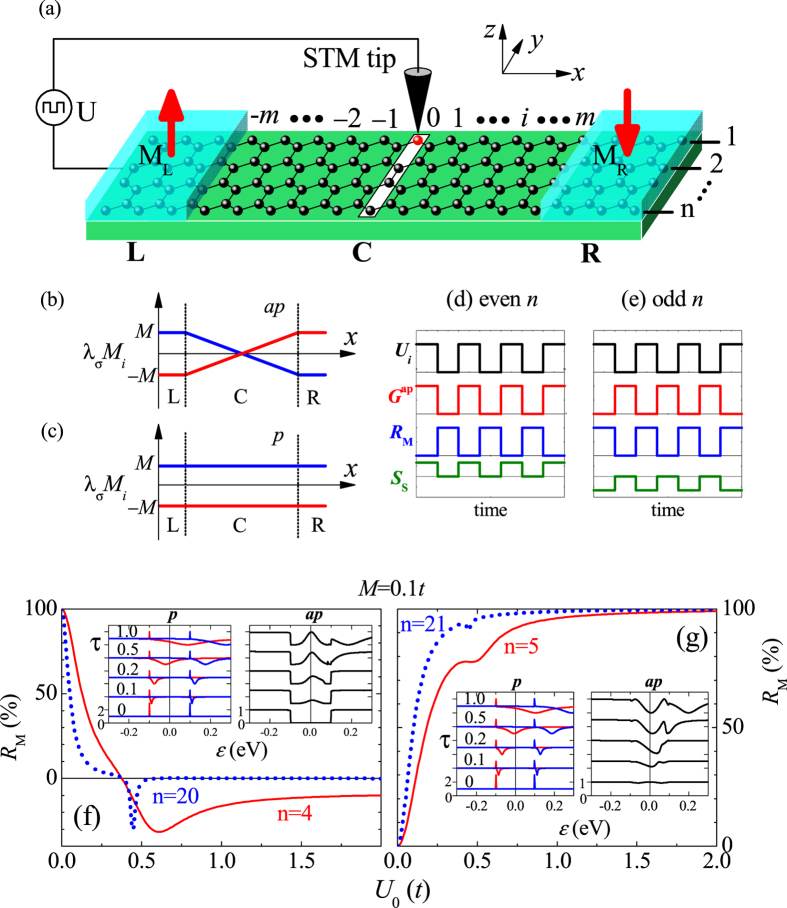
(**a**) Schematic structure of a FM *n*-ZNR two-probe junction with a central region of length 2 *m*. A third probe, the STM tip, can apply a local potential *U*_*i*_ at an edge site *i* ∈ [−*m*, *m*] to manipulate the magnetoresistance *R*_*M*_ and spin Seebeck coefficient *S*_S_. Edge site 0 (in red) has the minimal magnetization in the *ap* configuration. The colored arrow indicates the spin orientation of the electrodes. (**b,c**) Edge magnetization profile along the *x* direction in the *ap* and *p* configurations, respectively, for up (red) and down (blue) spins. (**d,e**) A possible variation set of 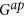, *R*_*M*_, and *S*_S_ with a square-wave signal *U*_*i*_ applied by the STM tip for even and odd *n*, respectively. (**f**) Zero temperature *R*_*M*_ versus *U*_0_ in a 4-ZNR (red) and a 20-ZNR (blue) with *m* = 5 and *M* = 0.1 *t*. The left inset shows the transmission spectra 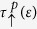 (red) and 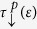 (blue) under *U*_0_ = 0, 0.1, 0.2, 0.5, 1.0 *t* (upward) in a 4-ZNR and the right shows 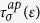. (**g**) Same as (f) for odd *n* = 5 (red) and 21 (blue) and the insets for *n* = 5. Note that the spectra for *U*_0_ ≠ 0 in the insets of (**f,g**) have been shifted upward for clearness and the vertical value of their fact parts is equal to 1.

As schemed in Fig. 1(a), we partition a FM ZNR into two-probe devices with left (L) and right (R) electrodes and a central (C) device region (domain wall in the *ap* configuration). A simplified tight-binding Hamiltonian can be used to describe the system^9^


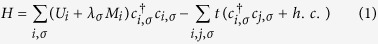


here 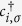 (*c*_*i*,*σ*_) is the creation (annihilation) operator of spin *σ* (↑ or ↓) on site *i*. *t* is the nearest-neighbor hopping integral and is chosen as the unit of energy throughout the paper. The uniform on-site energy of the corresponding pristine 2D materials gives the Dirac point energy and is set to zero. *U*_*i*_ and *λ*_*σ*_*M*_*i*_ (*λ*_↑_ = −1 and *λ*_↓_ = +1) are the on-site extrinsic potential energy from gate or disorder and the local magnetization, respectively. The magnetizations on the two edges are in parallel and linearly decay to zero from the edges to the center along the *y* direction. Along the *x* direction, *λ*_*σ*_*M*_*i*_ is constant *λ*_*σ*_*M* (full magnetization) in the electrodes and varies linearly in region C as depicted in Fig. 1(b,c) for the *ap* and *p* magnetic configurations of electrodes, respectively.

In the Landauer-Buttiker formalism with non-coherent effects neglected, the current of spin *σ* read 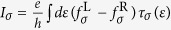 where 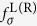 is the Fermi-Dirac distribution function 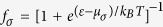 in electrode L (R). *T* and *μ*_*σ*_ are the electron temperature and the Fermi energy. *τ*_*σ*_(*ε*) = *Tr*[Γ_L_(*ε*)*G*^*r*^(*ε*)Γ_R_(*ε*)*G*^*a*^(*ε*)]_*σ*_ is the electron transmission calculated by nonequilibrium Green’s function (NEGF) method^32^. Here *G*^*r*^(*ε*) = [*G*^*a*^(*ε*)]^+^ = [*ε* − *h*_C_ − ∑_L_ − ∑_R_]^−1^ is the retarded Green’s function corresponding to the Hamiltonian *h*_*C*_ in region C and 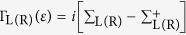 is the broadening function. The self-energy function ∑_L(R)_ due to the coupling between the device and electrode L (R) is obtained via the iterative procedure.

In the linear response regime of small voltage bias Δ*V*_*σ*_ and temperature difference Δ*T* between the electrodes, we express 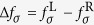 in Taylor expansion^33^ and have *I*_*σ*_ = *G*_0_*K*_0*σ*_(*μ*_*σ*_, *T*)Δ*V*_*σ*_ + *G*_0_*K*_1*σ*_(*μ*_*σ*_, *T*)Δ*T*/(*eT*) with *K*_*νσ*_ = ∫*dε(ε* − *μ*_*σ*_)^*ν*^*τ*_*σ*_(*ε*)(∂*f*_*σ*_/∂*ε*) for *ν* = 0, 1 and the conductance quantum *G*_0_ = *e*^2^/*h*. The tunneling magnetoresistance 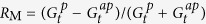 of the device is then evaluated^34^ from the total linear conductance 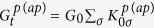 in the *p (ap*) configuration. The charge and spin Seebeck coefficients are defined by *S*_C(S)_ = (*S*_↑_ ± *S*_↓_)/2 with 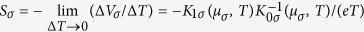 when *I*_*σ*_ = 0^35^,^36^,^37^,^38^. At low temperature the Mott formula^35^,^36^ 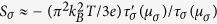 applies and analytical results can be obtained in simple cases.

More realistic Hamiltonians for ZNRs of specific materials can be obtained from the density functional theory (DFT) based on first-principles. As an example, we have carried out simulations on magnetoresistance and thermopower for doped *n*-ZGNRs with width *n* employing the Atomistic Toolkits (ATK) package^39^,^40^. The double-ζ plus polarization (DZP) basis set and an energy cutoff of 150 Ry within the local spin density approximation (LSDA) are used in the DFT simulation and a force tolerance of 0.03 eV/Å is set on each atom during the geometry relaxation. We find good agreement between results from the tight-binding model and from the first-principles simulations.

## Results and Discussion

We consider ZNR systems of *m* = 5 in the linear response regime with the Fermi energy at the neutral point, i.e. *μ*_*σ*_ = 0 throughout the manuscript. The effect of one single impurity on the Fermi energy is assumed negligible. For systems with Fermi energy away from the neutral point, the conductance of the system can be estimated from the energy dependence of transmission. In the *p* configuration, perfect ZNRs are uniformly magnetized and translationally symmetric along the *x* direction. The extended edge states have flat bands at the full magnetization energy *λ*_*σ*_*M* near the boundary of the first Brillouin zone. There is one transport channel for each spin *σ* at *ε* = 0^9^ so 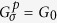 for both even and odd *n* at zero temperature. In the presence of a local extrinsic potential *U*_i_ at any edge site *i*, a bound state of energy 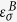 appears around the site with 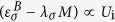. The bound state can slightly reduce 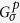 if 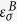 is close to zero^41^ as illustrated by the transmission spectra 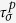 in the left insets of Fig. 1(f,g) where *U*_i_ > 0. The charge and spin thermopower are both very limited according to the Mott formula.

In the *ap* configuration the conductance becomes much more sensitive to *U*_i_ and *n*. We define the site *i* = 0 as the edge site where the magnetization reverses direction in the domain wall. For a geometrically left-right symmetric junction as schemed in Fig. 1(a), the residual magnetization vanishes at site 0, i.e. *M*_0_ = 0. In the absence of extrinsic potential *U*_i_, we have 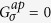 for even *n* and 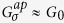 for odd *n* at zero temperature due to geometry symmetry as indicated in the right insets of Fig. 1(f,g). The corresponding magnetoresistance *R*_*M*_ is about 100% for even *n* and very small for odd *n*^10^,^11^,^12^,^13^,^17^. In the presence of *U*_i_, we will present the results for *U*_i_ > 0 in two typical cases, *i* = 0 and *i* > 0 in the following since those for *U*_i_ < 0 or *i* < 0 can be then deduced based on the symmetry of the systems.

At first we apply a local potential *U*_0_ only at edge site 0 of an *n-*ZNR. For even *n*, as shown in the right inset of Fig. 1(f), a transmission peak emerges at *ε* = 0 with 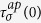 approaching to unit as *U*_0_ increases. The corresponding *R*_*M*_, as plotted in Fig. 1(f), then decreases from 100% to near zero and can become negative if 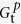 is reduced to 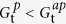 by the bound state. In contrast, for odd *n* as illustrated in Fig. 1(g), 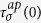 decreases inversely with *U*_0_ from unit and approaches to zero, which leads to a jump of *R*_*M*_ from zero to near 100%. When the dip bottom of 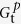 due to the bound state passes through the Fermi energy near *U*_0_ = 0.5 *t*, a *R*_*M*_ minimum appears for both even and odd *n*.

**Figure 2:**
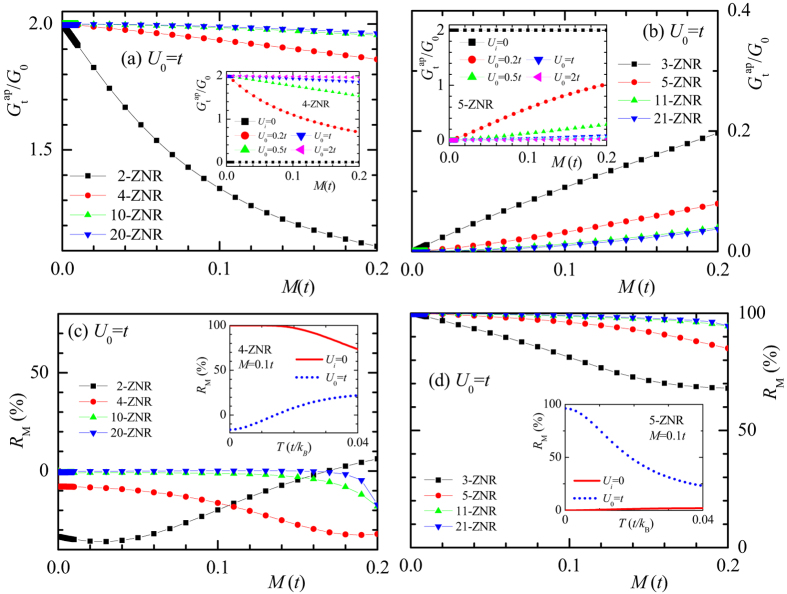
(**a**,**b)** Show the total zero-temperature conductance 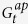 versus the edge magnetization *M* in the presence of potential *U*_0_ = *t* at edge site 0 of *n*-ZNRs for even *n* (2, 4, 10, 20) and odd *n* (3, 5, 11, 21), respectively. The corresponding magnetoresistance *R*_*M*_ is illustrated in (**c**,**d**). 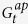 versus *M* and *R*_*M*_ versus *T* at *M* = 0.1 *t* are plotted in the insets of the upper and lower panels, respectively, for 4-ZNRs and 5-ZNRs under different *U*_0_. Note that results of *M* = 0 are not shown.

The conductance and magnetoresistance are also relevant to the full magnetization *M* which can vary for different materials and/or substrates. In Fig. 2(a,b), we plot 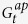 versus *M* under *U*_0_ = *t* for various even and odd *n*, respectively. In nonmagnetic ZNRs (*M* = 0), 
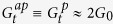
 or *R*_*M*_ = 0. As *M* increases, 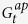 decreases gradually from 2*G*_0_ but remains high for even *n.* In contrast, for odd *n*, 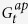 is very sensitive to *M* and jumps down to zero once *M* becomes finite, similar to the behavior of perfect even-width ZNRs^10^ as shown in the inset of Fig. 2(a). It then increases monotonically with *M* but remains in low values. In the range of *M* ∈ [0, 0.2]*t*, 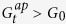 in 2-ZNRs while 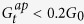 in 3-ZNRs and the difference between 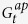 of even- and odd-width ZNRs enlarges as the width increases. In the insets of Fig. 2(a,b), we plot also the variation of 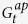 versus *M* under *U*_0_ of different strengths to show its dependence. At any finite *M*, 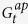 increases (decreases) from zero (2*G*_0_) and saturates to 2*G*_0_ (zero) for even (odd) *n* as *U*_0_ increases (see also the insets of Fig. 1(f,g) and Figure S1).

The corresponding magnetoresistances *R*_*M*_ versus *M* are shown in Fig. 2(c,d) for even and odd *n*, respectively. Under *U*_0_ = *t*, *R*_*M*_ is well below 10% for all even *n* and above 60% for all odd *n* in the range *M* ∈ [0, 0.2]*t*. When the energy of the bound state confined by *U*_0_ in the *p* configuration is close to the Fermi energy, *R*_*M*_ can be negative for even *n* and shows an extra dip for odd *n*.

Using the density functional theory combined with the nonequilibrium Green’s function implemented in the ATK package^39^,^40^, we have simulated the transmission spectrum of hydrogen-passivated 4-ZGNRs and 5-ZGNRs substitutionally doped by a boron atom at edge site 0. The result agrees well with that from the tight-binding model of parameters *M* = 0.07 *t*, *U*_0_ = 1.15 *t* for 4-ZGNR and *M* = 0.08 *t*, *U*_0_ = 1.20 *t* for 5-ZGNR with *t* = 2.7 eV as shown in Figure S2. The magnetoresistance switch effect can be quite robust in real materials like ZGNRs. In the insets of Fig. 2(c,d), we plot *R*_*M*_ versus the temperature T for 4-ZNRs and 5-ZNRs with parameters close to those of the above boron doped ZGNRs, i.e. *U*_0_ = *t* and *M* = 0.1 *t*. The behaviors of perfect/doped and even/odd ZNRs remains well distinguished from each other up to the room temperature *T* = 0.01 *t*/*k*_*B*_.

In the simplest case of 2-ZNR structure with *m* = 0, we have the analytical transmission expressions 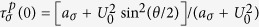 and 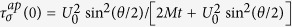 with 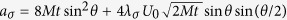 and 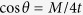. They may be helpful for qualitative analysis in more general contexts. For *M* = 0.1 *t*, the zero temperature magnetoresistance decreases from *R*_*M*_ = 100% at *U*_*i*_ = 0 to *R*_*M*_ = −22.1% at *U*_0_ = *t* similar to the result presented in Fig. 2(c) with *m* = 5 (see Figure S3 for the details).

If the potential shifts from site 0 to a site *i* > 0 where a downward residual magnetization *M*_*i*_ exists, the two-fold rotation symmetry of the system is broken and the transmission becomes spin dependent. The spin thermopower in the *ap* configuration can be greatly enhanced and shows strong even-odd effects on the nanoribbon width.

**Figure 3:**
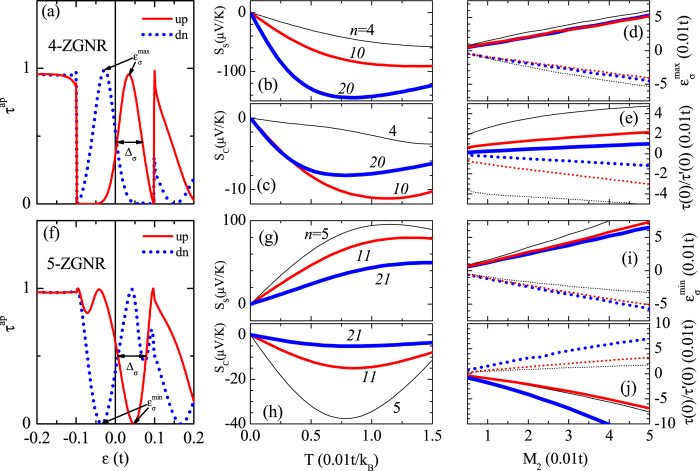
(**a**) 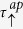 (solid) and 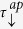 (dotted) of a 4-ZNR under a potential *U*_2_ = *t* at edge site 2. (**b**,**c**) *S*_S_ and *S*_*C*_ versus the temperature *T*, respectively, of *n*-ZNRs for even *n* = 4 (thin), 10 (medium), and 20 (thick) at *U*_2_ = *t* and *M* = 0.1 *t*. (**d**) 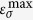 and (e) *τ*_*σ*_(0)/*τ*′_*σ*_(0) of up (solid) and down (dotted) spins are plotted versus the local edge magnetization *M*_2_ for *n* = 4 (thin), 10 (medium), and 20 (thick) as *M* varies. (**f–j**) The same as in (**a–e**) for odd *n* = 5, 11, 21 with 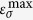 replaced by 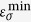.

In even-width nanoribbons the spin-up (down) transmission peak shifts accordingly from 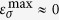 to 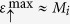 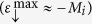 as shown in Fig. 3(a) for *U*_2_ = *t*, *n* = 4, *i* = 2, and *M*_2_ = *iM*/(*m* + 1) = 0.033 *t* with *M* = 0.1 *t* and *m* = 5. The transmission spectrum then has a positive 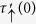 (negative 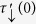) which results in a negative *S*_↑_ (positive *S*_↓_) at low temperature according to the Mott formula. Interestingly, the spin Seebeck coefficient appears usually much greater than the charge one, i.e. |*S*_*S*_| ≫ |*S*_*C*_|, because the transmission peaks of up and down spins are located almost symmetrically beside the Fermi energy, i.e. 
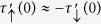
 and *τ*_↑_(0) ≈ *τ*_↓_(0). *S*_*S*_ can be further enhanced in wider nanoribbons where the transmission peaks become sharper with bigger |*τ*′(0)| and smaller *τ*(0).

The temperature dependent *S*_*S*_ and *S*_*C*_ plotted in Fig. 3(b,c), respectively, indicate that the Mott formula is valid below the critical temperature *T*_*c*_ = *τ*(1−*τ* )/*τ*′*k*_*B*_|_*ε*=0_ which is determined by the width Δ_*σ*_ and the position 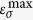 of the transmission peak. At high temperature the Seebeck coefficients decay gradually with the temperature due to the nonlinearity of the transmission spectrum.

The Seebeck coefficients can also be manipulated by the full magnetization *M* since both 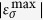 and Δ_*σ*_ increase with *M*. As illustrated in Fig. 3(d,e) for *i* = 2, the variation of *τ*(0) is limited due to the opposite effects from increase of 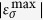 and Δ_*σ*_ but |*τ*′(0)| decreases quickly and results in the decrease of *S*_*σ*_. On the other hand, if the potential shifts from site 2 to site 3 while *M* remains fixed, 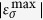 increases since *M*_3_ > *M*_2_, *S*_*S*_ then increases with the decrease of *τ*(0).

In odd-width nanoribbons, the spin-up (down) transmission dip shifts from 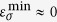 to 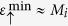 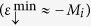 when the potential moves from site 0 to a site *i* > 0 as shown in Fig. 3(f) for *n* = 5. The dip width Δ_*σ*_ increases with 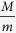 but decreases with *n*. We have 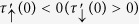 and then a positive *S*_*S*_ rather than the negative one in even-width nanoribbons. *S*_*S*_ decreases with *i* since the corresponding increase of 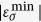 enhances *τ*(0). *S*_*S*_ decreases also with *M* because the increase of both Δ_*σ*_ and 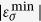 reduces *τ*′(0) as shown in Fig. 3(j). If *n* increases, *S*_*S*_ becomes weaker further as illustrated in Fig. 3(g) when the decrease of Δ_*σ*_ leads to competing larger *τ*′(0) and larger *τ*(0). Different from the cases in even-width ZNRs, as shown in Fig. 3(i,j), 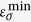 and 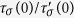 are more sensitive to *n* in odd-width ZNRs.

## Summary

Zigzag nanoribbons of graphene-like materials are expected very useful for spintronics due to their edge spin polarization or magnetism. Giant magnetoresistance exists in pristine even-width ferromagnetic nanoribbons. With the help of an atomistic gate potential or edge disorder, the giant magnetoresistance can be switched off (on) in even- (odd-) width nanoribbons. This originates from the jump of electronic transmission at the Fermi energy from zero to near 100% or verse vise in the antiparallel magnetic configuration if the potential is located at the transition interface of magnetization. If the potential shifts from the interface, the transmission peaks or dips of opposite spins split symmetrically beside the Fermi energy. The spin thermopower then becomes very large according to the Mott formula showing strong magneto-thermopower effect. This suggests that spin current can be produced from temperature gradient in the material.

## Additional Information

**How to cite this article**: Zhai, M.-X. and Wang, X.-F. Atomistic switch of giant magnetoresistance and spin thermopower in graphene-like nanoribbons. *Sci. Rep.* **6**, 36762; doi: 10.1038/srep36762 (2016).

**Publisher’s note:** Springer Nature remains neutral with regard to jurisdictional claims in published maps and institutional affiliations.

## Supplementary Material

Supplementary Information
