# Supplementary material for: Atomistic switch of giant magnetoresistance and spin thermopower in graphene-like nanoribbons
Source: Sci Rep. 2016 Nov 18;6:36762. doi: 10.1038/srep36762 (PMC5114670; doi:10.1038/srep36762)
Supplement: Supplementary Information [file srep36762-s1.pdf]

Supplementary materials

Atomistic switch of giant magnetoresistance in graphene-like nanoribbons

Ming-Xing Zhai and Xue-Feng Wang

S1. Detailed transmission spectrum

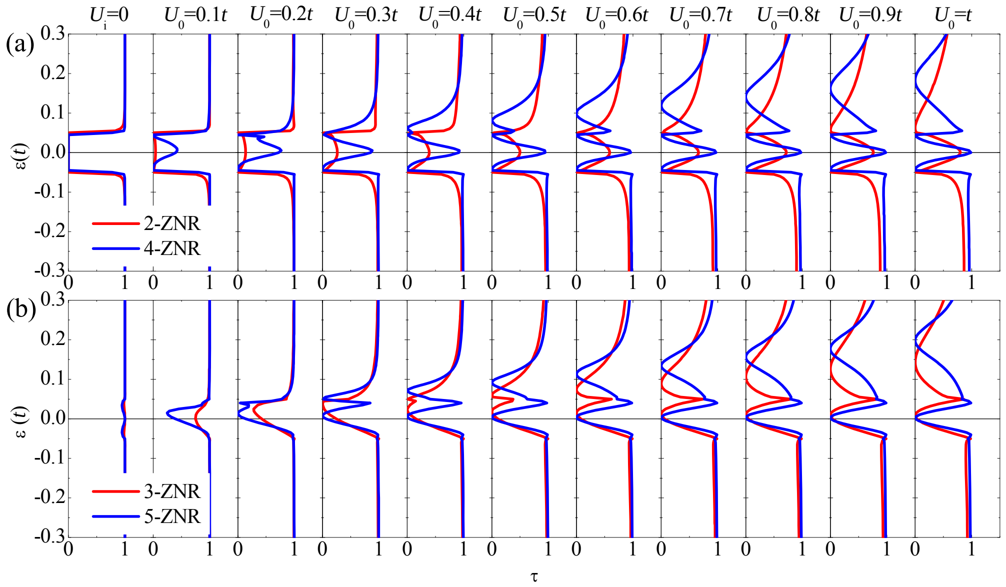

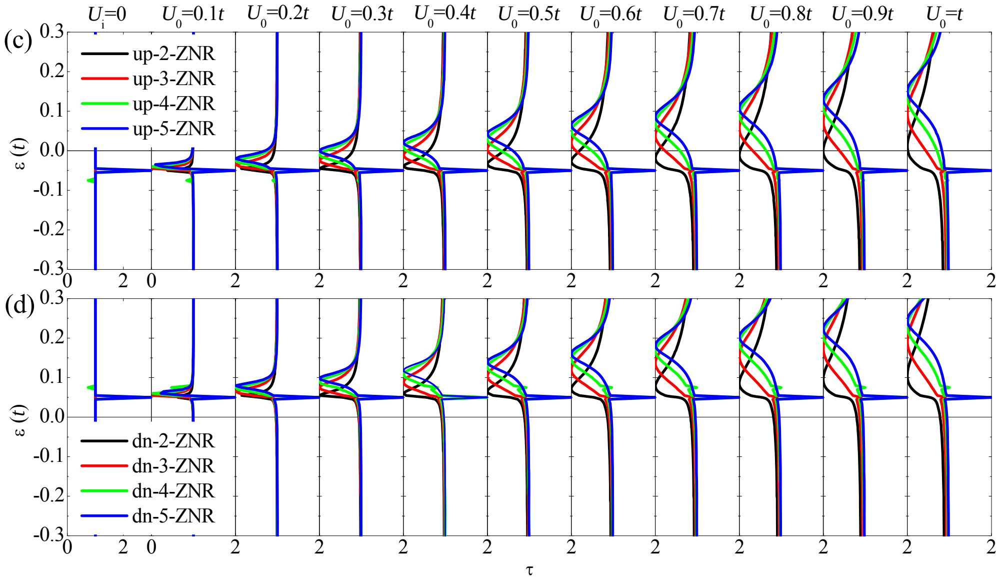

Supplementary Figure S1. Transmission spectra of pristine *n*-ZNRs and those with a local potential U_0 at edge site 0 in cases: (a) *n*=2, 4 and (b) *n*=3, 5 in the *ap* configuration; (c) spin-up and (d) spin-down spectra in the *p* configuration for n=2, 3, 4, and 5. The full magnetization energy is $M = 0.05t$.

S2. Comparison between results of tight-binding model and DFT simulation

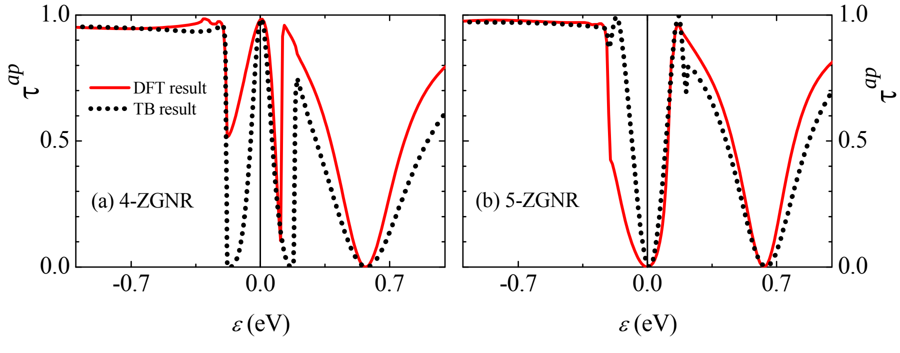

Supplementary Figure S2. The transmission spectra of *n*-ZGNRs edge doped by one boron atom at site 0 in the *ap* configuration obtained from *ab initio* DFT simulations (solid) are compared with those from the tight-binding model (dotted). The tight-binding parameters are (a)

$M = 0.07t$, $U_0 = 1.15t$ for n=4 and (b) $M = 0.08t$, $U_0 = 1.20t$ for n=5. Our simulation of transport properties are carried out using the Atomistix Toolkits (ATK) package based on the density functional theory (DFT) combined with the non-equilibrium Green's function (NEGF) method. We employ an exchange-correlation functional in the local spin density approximation with the Perdew-Zunger (PZ) parameters, a real space cutoff energy of 150 Ry, a double-ζ polarization linear-combination-of-atomic orbital basis set for all atoms, and a k-point sampling of 1×1×100 grid in the 1D Brillouin zone. A vacuum layer of 15Å wide is inserted between edges and planes of the ZGNRs in the supercell. The Newton method with a force tolerance of 0.03eV/Å was used for the geometric optimization.

S3. Analytical derivation of magnetoresistance in 2-ZNRs

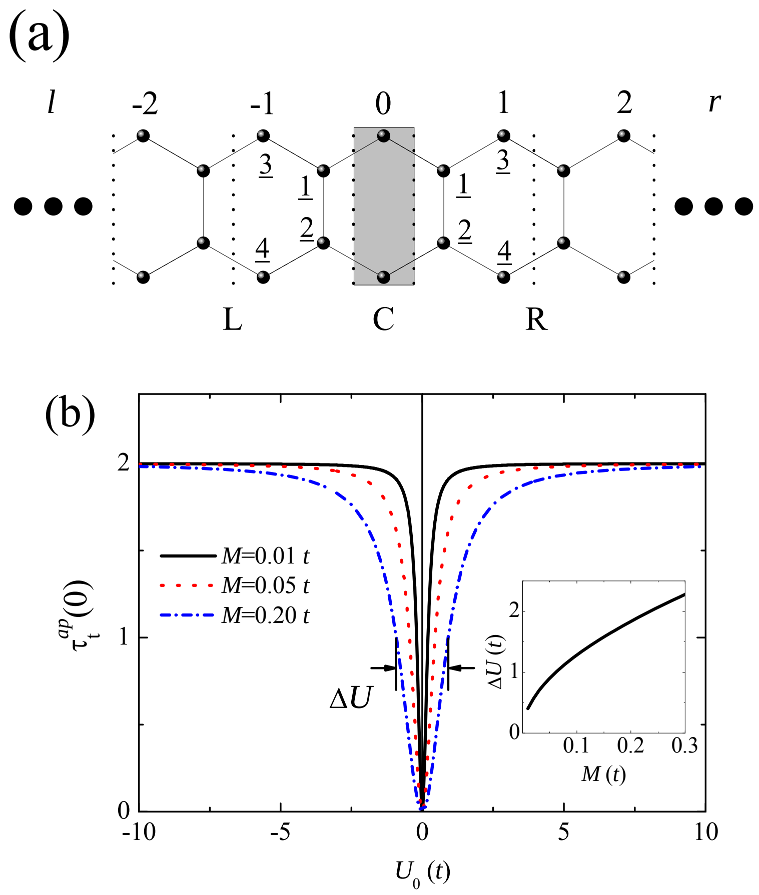

Supplementary Figure S3. (a) A simple 2-ZNR two-probe system with two atoms in the central region (grey shadow). (b) The Fermi energy transmission $\tau_t^{ap}(0)$ versus the potential U_0 at site $i=0$ in the above 2-ZNR system of different full magnetizations M . The half-height width of the transmission dip at the Femi energy ΔU versus M is plotted in the inset.

For spin-up electrons in a FM 2-ZNR two-probe system of $m=0$ as schemed in Fig. S3(a), the tight-binding Hamiltonian of left electrode $\hat{H}_{\mathrm{L}}$ has tridiagonal nonzero elements

$$H_{l,l}=\begin{pmatrix} 0 & -t & -t & 0 \\ -t & 0 & 0 & -t \\ -t & 0 & \Delta_{\mathrm{L}} & 0 \\ 0 & -t & 0 & \Delta_{\mathrm{L}} \end{pmatrix} \text{ and } H_{l,l+1}=\begin{pmatrix} 0 & 0 & 0 & 0 \\ 0 & 0 & 0 & 0 \\ -t & 0 & 0 & 0 \\ 0 & -t & 0 & 0 \end{pmatrix} \tag{s1}$$

with the primitive cell index $l\in(-\infty,-1]$, the nearest neighbor coupling parameter $-t$ and the on-site energy Δ_{L} due to electrode magnetization. $\Delta_{\mathrm{L}}=-M$ in the *ap* magnetic configuration. For the right electrode, we have Hamiltonian $\hat{H}_{\mathrm{R}}$ similar to $\hat{H}_{\mathrm{L}}$ but $\Delta_{\mathrm{L}}=M$ in the *ap* configuration.

If there is no residual magnetization on site 0 in the *ap* configuration, the Hamiltonian of the central region is $\hat{H}_{\mathrm{C}}=H_{00}=\begin{pmatrix} U_0 & 0 \\ 0 & 0 \end{pmatrix}$ and the Green's function at the Fermi energy in the central region reads $G_{\mathrm{C}}^{ap}(0)=(-H_{00}-\Sigma_{\mathrm{L}}-\Sigma_{\mathrm{R}})^{-1}$. The spin-up transmission between the electrodes in the *ap* configuration is then obtained as

$$\tau_{\uparrow}^{ap}(0)=tr\left(\Gamma_{\mathrm{L}}^{ap}G_{\mathrm{C}}^{ap}\Gamma_{\mathrm{R}}^{ap}G_{\mathrm{C}}^{ap\dagger}\right)\Big|_{\varepsilon=0}=\frac{U_0^2\sin^2\frac{\theta}{2}}{2Mt+U_0^2\sin^2\frac{\theta}{2}} \quad . \tag{s2}$$

The spin-down transmission can be obtained in the same way and we have $\tau_{\downarrow}^{ap}(0)=\tau_{\uparrow}^{ap}(0)$. The total transmission $\tau_{t}^{ap}(0)=\tau_{\uparrow}^{ap}(0)+\tau_{\downarrow}^{ap}(0)$ is zero if the potential $U_0=0$ and approaches to two when $U_0^2>>Mt$. In Fig. S3 (b), we plot the transmission $\tau_{t}^{ap}(0)$ versus potential U_0 for various magnetizations M. The curve has a dip at $U_0=0$ with half depth $\tau_{t}^{ap}(0)=1$ at $U_0=\pm4\sqrt{M/(4t-M)}$. The half depth width of the dip is $\Delta U=8t\sqrt{M/(4t-M)}\approx2\sqrt{Mt}$ for small $M<<t$.

In p configuration, we have $\hat{H}_{\uparrow C}^{p}=\begin{pmatrix}-M+U_0 & 0\\ 0 & -M\end{pmatrix}$ and $\hat{H}_{\downarrow C}^{p}=\begin{pmatrix}M+U_0 & 0\\ 0 & M\end{pmatrix}$ for spin-up and spin-down electrons, respectively. The total transmission reads

$$\tau_t^p=\frac{a_\sigma+U_0^2\sin^2\dfrac{\theta}{2}}{a_\sigma+U_0^2}=\frac{e^2}{h}\left(1-\frac{U_0^2\left(\cos\theta+1\right)/2}{a_\sigma+U_0^2}\right)\qquad\text{(s3)}$$

with $a_\sigma=8Mt\sin^2\theta+4\lambda_\sigma U_0\sqrt{2Mt}\sin\theta\sin(\theta/2)$, $\lambda_\uparrow=-1$, and $\lambda_\downarrow=+1$.
